# Severe midaortic syndrome: a stepwise approach to treatment with drug-eluting balloons: a case report

**DOI:** 10.1093/ehjcr/ytz017

**Published:** 2019-02-25

**Authors:** Peter Zartner, Christopher Hart, Martin B E Schneider

**Affiliations:** Department of Cardiology, German Paediatric Heart Center, Arnold Janssen Str. 29, Sankt Augustin, Germany

**Keywords:** Coarctation, Arterial hypertension, Non-surgical interventional treatment, Congenital heart disease, Case report, Drug-eluting balloon

## Abstract

**Background:**

Severe forms of the midaortic syndrome cause persistent arterial hypertension and can lead to angina abdominalis. Untreated, morbidity, and mortality are significant. In addition to palliation through bypass surgery, few other therapeutic approaches lead to a long-term relief. Drug-eluting balloons (DEB) covered with paclitaxel, a cytostatic drug, have proven to be effective in bifurcational lesions and for in-stent stenoses in coronary arteries.

**Case summary:**

In a 15-year-old girl with severe midaortic syndrome and multiple collateral arteries, four interventional balloon dilatations with DEB of increasing diameters resolved the stenosis within 8 months. After a procedure free interval of now 2.7 years, the anatomical and the physical condition of the patient remained unchanged.

**Discussion:**

This stepwise approach, with a low procedural risk and a lasting result may justify further investigations into this combined treatment.

**Learning points**

In patients with a midaortic syndrome:

- The stenosis may only be found below the diaphragm.
- Involvement of the renal arteries can cause severe arterial hypertension.
- Stepwise dilation with a drug-eluting balloon is an option to treat.

## Introduction

Interventional dilation with drug-eluting balloons (DEB) is an established therapeutic option in coronary and peripheral arterial disease in adults.^1^ With successful results in adults, the use in paediatric patients with congenital stenosis has begun. Besides a few clinical trials in pulmonary vein stenosis,^2^ only a single case with the use of a paclitaxel eluting balloon in a child with Takayasu’s disease and renal artery stenosis has been published.^3^

The midaortic syndrome is a rare diagnosis and is defined as segmental narrowing of the proximal abdominal aorta. It can be acquired or congenital. The reduced blood perfusion to the renal arteries causes severe renovascular hypertension. Effective therapeutic options are surgical with different modifications of aortoaortic bypass grafts^4^^,^^5^ or interventional with the implantation of stents,^6^^,^^7^ but results are not always satisfactory, particularly on long-term follow-up.

## Timeline

**Table:** 

Date	Procedures	Time difference
10 February 2015	*Suspected diagnosis* because of syncope, episodes of headache, nose bleeding, and blood pressure difference between arms and legs.	Age 15 years
	*Diagnosis* by echocardiography with left ventricular hypertrophy and a narrow long segment of the abdominal aorta proximal to and involving the renal arteries	
12 February 2015	Dilation with 7 mm drug-eluting balloons (DEB)	2 days
19 April 2015	Dilation with 8 mm DEB	9.4 weeks
22 June 2015	Dilation with 10 mm DEB	9.1 weeks
28 October 2015	Dilation with 12 mm DEB	18.2 weeks
20 June 2018	Control magnetic resonance imaging demonstrated no evidence of recurrence of stenosis. The blood pressure was 139/64 mmHg at the right arm and 127/65 mmHg at the right leg under therapy with atenolol and amlodipine.	2.64 years

## Case presentation

**Figure 1:**
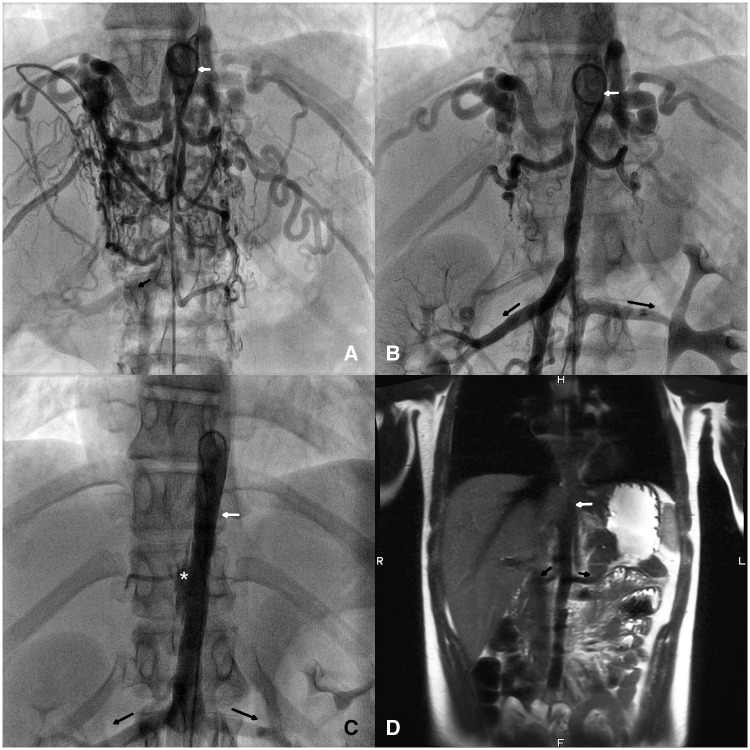
(*A*) Initial presentation of the abdominal aorta with subtotal occlusion caused by a 1.7 mm catheter passing through the stenosis. There is a massive collateralization and only minimal contrast opacification of the right renal artery. The white arrow marks the upper entrance to the tubular coarctation of the abdominal aorta, and the black arrows mark the course of the left and right renal artery, if visible. (*B*) Anatomy after the first dilation with a Paclitaxel covered balloon. (*C*) After the fourth intervention using a 12 mm drug-eluting balloon an endothelial lesion and intra mural contrast was noted (white asterisk). (*D*) Magnetic resonance imaging at latest follow-up 2.7 years after this last intervention shows an unobstructed aorta without collateralization.

A 15-year-old female patient (bodyweight 50 kg, height 160 cm) presented with syncope. Severe arterial hypertension was diagnosed (178/147 mmHg left arm, 102/83 mmHg left leg). She reported previous episodes of exercise independent headaches and nose bleeds. Auscultation revealed no cardiac murmurs, but an accentuated second heart sound and reduced pulses in the lower limbs. There were no clinical signs to suggest Alagille or William’s syndrome or clinical evidence of neurofibromatosis. Blood tests showed normal inflammatory markers and normal creatinine. The urine dip showed no markers for blood or protein. No regular medication was taken at the time she presented. The electrocardiogram showed normal sinus rhythm, no significant signs of left ventricular (LV) hypertrophy or abnormal repolarization. Echocardiography detected a severe concentric LV hypertrophy (14 mm diastolic septal diameter, 15 mm diastolic diameter of the LV posterior wall) and a long hypoplastic segment in the abdominal aorta with massive arterial collateralization. Cardiac catheterization confirmed the diagnosis of a midaortic syndrome with a minimum diameter of <1.5 mm and a gradient of 50 mmHg between the aortic arch and the femoral arteries (*Figure 1A*). The narrow part began directly under the diaphragm above the truncus coeliacus and continued down to the bifurcation of the arteriae iliacae including the renal arteries, with reversed flow in the caudal aorta up to the renal arteries. Because of the extraordinary length of the hypoplastic segment of 23 cm, we decided for a stepwise interventional therapy with a paclitaxel covered balloon. No additional computed tomography scan or magnetic resonance imaging (MRI) was performed. Consultation with our surgeon, supported the interventional approach. The proposed procedure was reviewed and approved by our institutional review board, extensively discussed with the patient’s family, and informed consent was obtained before proceeding.

After balloon interrogation of the long stenotic abdominal aorta using a 6 × 20 mm Tyshak balloon (NuMED Inc., Hopkinton, NY, USA) to differentiate between rigid stenosis and hypoplastic parts, serial balloon dilation with a 7 × 40 mm paclitaxel covered In.Pact balloon (Medtronic, Minneapolis, MN, USA) (3 µg/mm^2^ Paclitaxel on the balloon) was performed (*Figure 1B*) from end to end of the stenosis. Planned redilations, to stepwise improve the aortic compliance and reduce the risk of dissection, were performed after 2, 4, and 8 months with Elutax balloons ranging from 8 mm to 12 mm diameter (Aachen Resonance, Düsseldorf, Germany) *(2 µg/mm^2^ Paclitaxel).* At the end of the last interventional procedure, a mild endothelial lesion at the former narrowest point of the aorta was noticed (*Figure 1C*). This lesion healed and after 2.7 years MRI showed a stable and adequate result (*Figure 1D*). Today the girl is in unrestricted physical condition. The blood pressure has markedly improved (139/64 mmHg right arm and 127/65 mmHg right leg) under therapy with atenolol and amlodipine.

The patient and patient’s family consented to the publication of this case’s history and the images presented.

## Discussion

The midaortic syndrome is rare, but its severe form, with involvement of the renal arteries, is associated with significant morbidity and mortality.^8^ The results of routine measurement of the blood pressure in children should raise suspicion. Because of the non-causal treatment using antihypertensive drug combinations and poor long-term results in endovascular intervention, surgical treatment is widely accepted as the treatment of choice.^4^^,^^9^ However, the implantation of an aortoaortic bypass can become a very complex operation with a high intraoperative risk,^5^ frequently followed by the need for re-interventions.

If the midaortic syndrome is interpreted as a local aortic wall disease, a combined medical and endovascular treatment might be a reasonable therapeutic option. Drug-eluting balloons and stents have proven to be safe and effective in coronary artery diseases. Paclitaxel, a cytostatic drug, which is lipophilic, anti-proliferative and irreversibly stops the cell division between meta- and Ana-phase is widely used to cover DEB. In children some reports showed a minor and normally tolerable drug-toxicity of paclitaxel after local therapy.^2^^,^^10^

In our patient, the hypoplastic segment of the aorta was very long and included all origins from the coeliac trunk to the iliac vessels. Survival was only possible because of massive collateralization. Using stents in this setting, overstenting of nearly all side branches would have been unavoidable. Surgical therapy with an aortic bypass graft of this long segment would have generated a high risk because of endangered vascular supply of all abdominal organs. Endovascular treatment with a DEB to enlarge the aortic diameter and reduce the risk for re-stenoses was seen as the best therapeutic option in this setting. As the initial result was promising, repeated re-dilations with a stepwise increasing diameter of the DEB were justified.

The latest measured residual gradient of <10 mmHg could not prevent from a persistent arterial hypertension as known from late diagnosed coarctation, so antihypertensive medication was required.

## Conclusion

This is a single case experience with an intervention-free clinical follow-up and an adequate local result. Repeated balloon dilations with paclitaxel eluting balloons have demonstrated to be an effective and safe therapeutic option in this patient with a severe and long segment midaortic syndrome. This treatment needs to be applied in comparable situations to better judge its value for similar patients.

**Slide sets:** A fully edited slide set detailing this case and suitable for local presentation is available online as Supplementary data.

**Consent:** The author/s confirm that written consent for submission and publication of this case report including image(s) and associated text has been obtained from the patient in line with COPE guidance.

**Conflict of interest:** none declared.

## Supplementary Material

Supplementary Data
