# Supplementary material for: Severe midaortic syndrome: a stepwise approach to treatment with drug-eluting balloons: a case report
Source: Eur Heart J Case Rep. 2019 Feb 25;3(1):ytz017. doi: 10.1093/ehjcr/ytz017 (PMC6439396; doi:10.1093/ehjcr/ytz017)
Supplement: Supplementary Data [file ytz017_supp.pptx]

## Slide 1
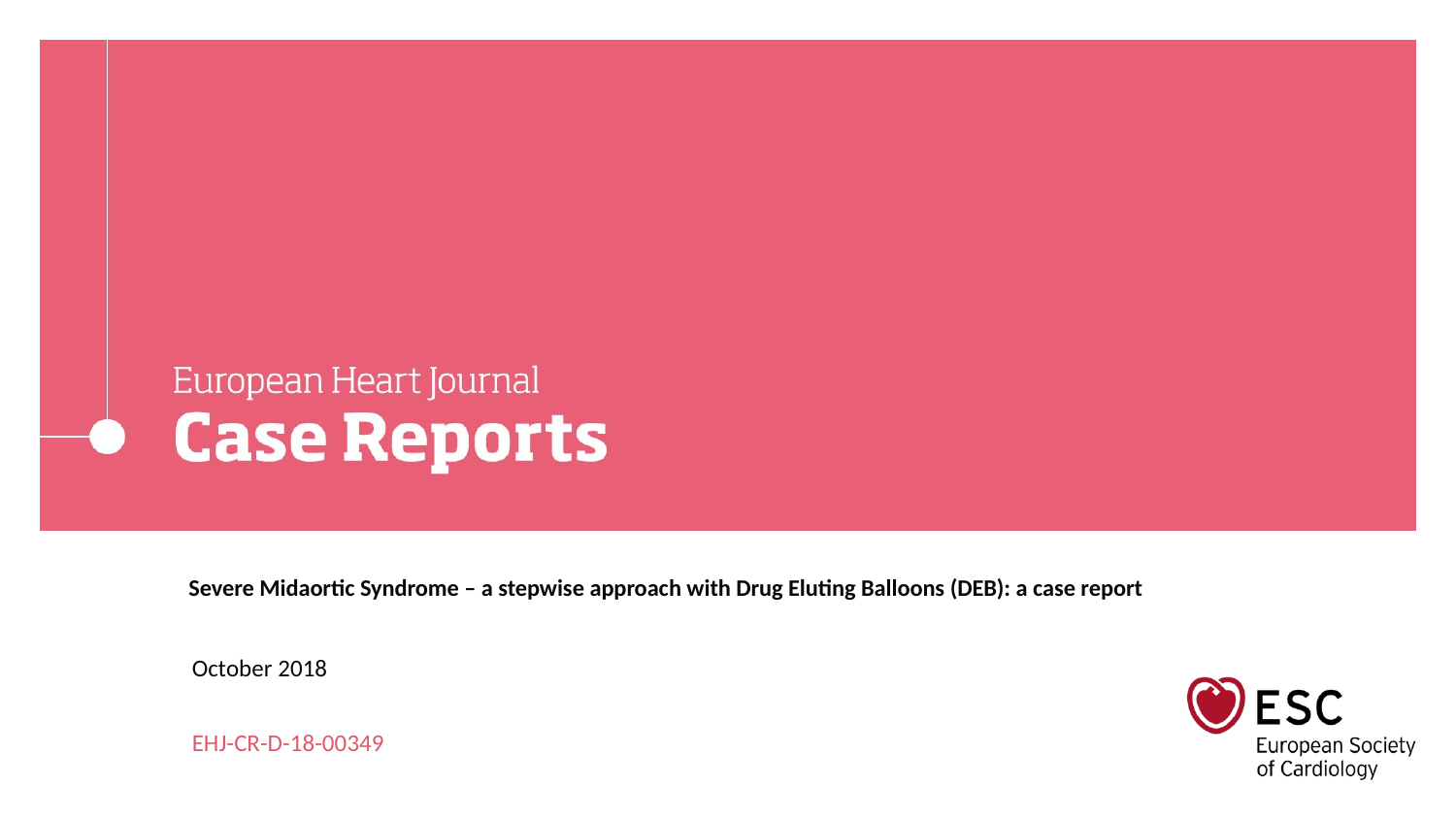

# Severe Midaortic Syndrome – a stepwise approach with Drug Eluting Balloons (DEB): a case report
October 2018
EHJ-CR-D-18-00349

## Slide 2
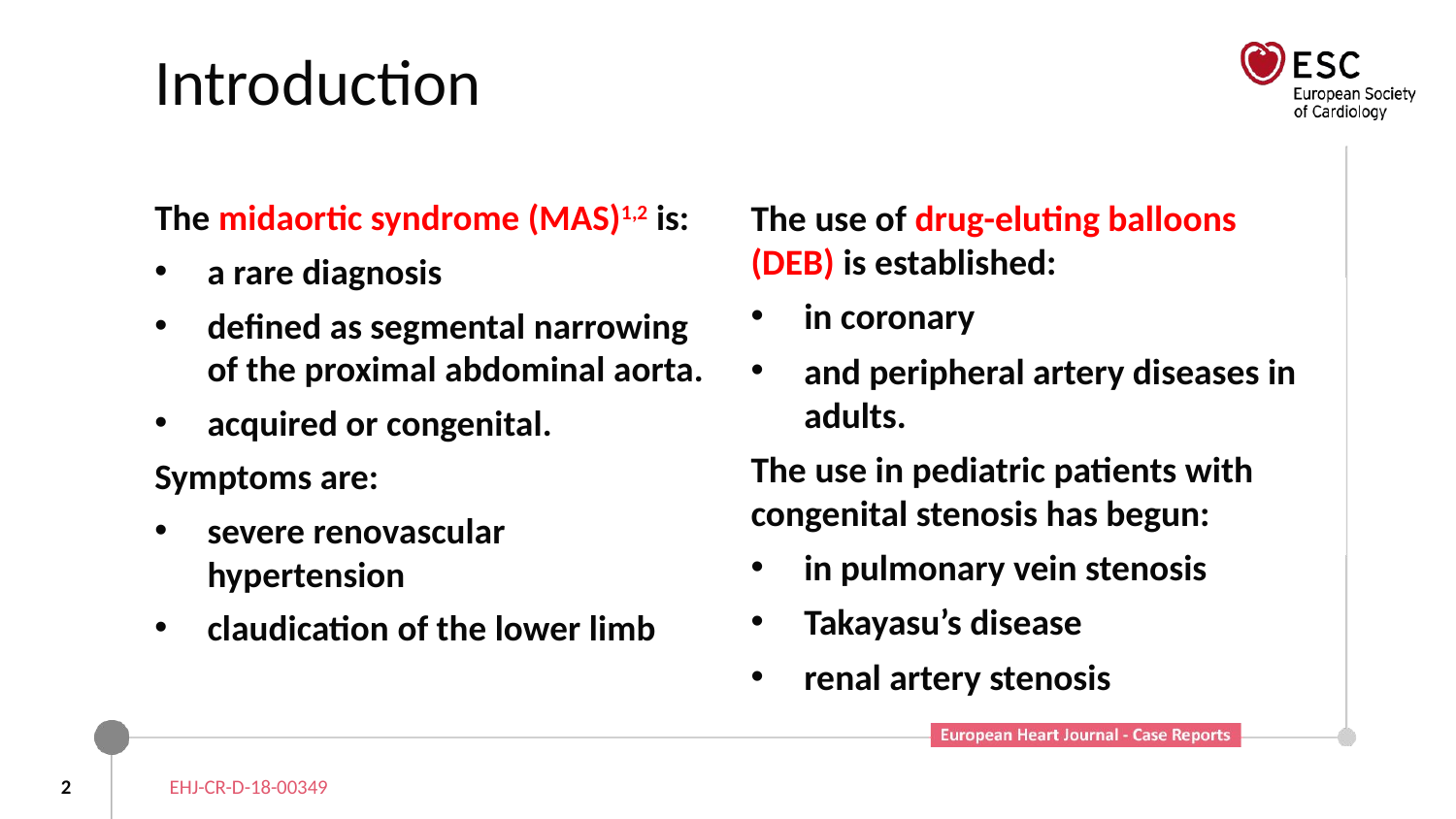

# Introduction
The midaortic syndrome (MAS)1,2 is:
a rare diagnosis
defined as segmental narrowing of the proximal abdominal aorta.
acquired or congenital.
Symptoms are:
severe renovascular hypertension
claudication of the lower limb
The use of drug-eluting balloons (DEB) is established:
in coronary
and peripheral artery diseases in adults.
The use in pediatric patients with congenital stenosis has begun:
in pulmonary vein stenosis
Takayasu’s disease
renal artery stenosis
2
EHJ-CR-D-18-00349

## Slide 3
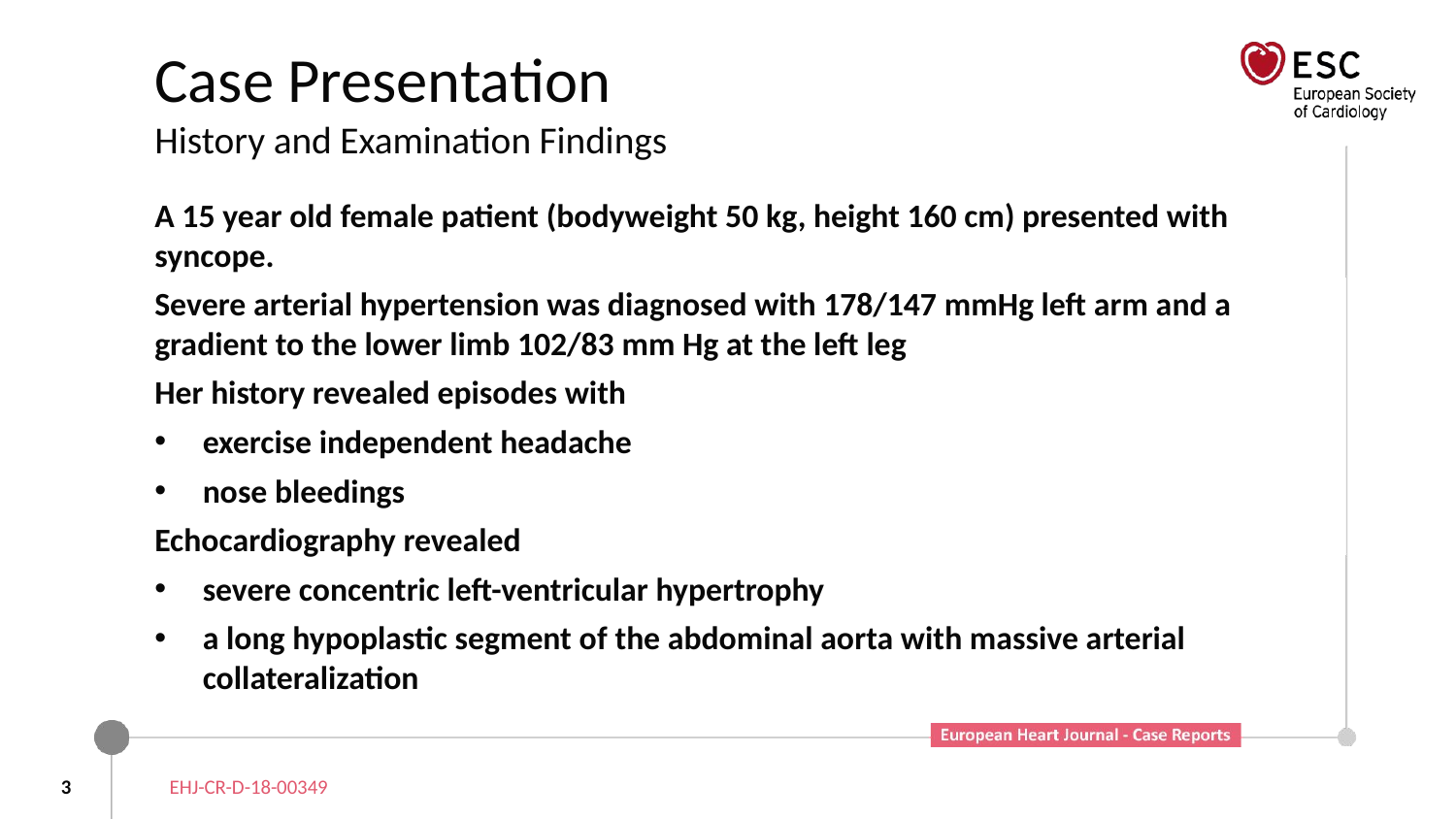

# Case Presentation History and Examination Findings
A 15 year old female patient (bodyweight 50 kg, height 160 cm) presented with syncope.
Severe arterial hypertension was diagnosed with 178/147 mmHg left arm and a gradient to the lower limb 102/83 mm Hg at the left leg
Her history revealed episodes with
exercise independent headache
nose bleedings
Echocardiography revealed
severe concentric left-ventricular hypertrophy
a long hypoplastic segment of the abdominal aorta with massive arterial collateralization
3
EHJ-CR-D-18-00349

## Slide 4
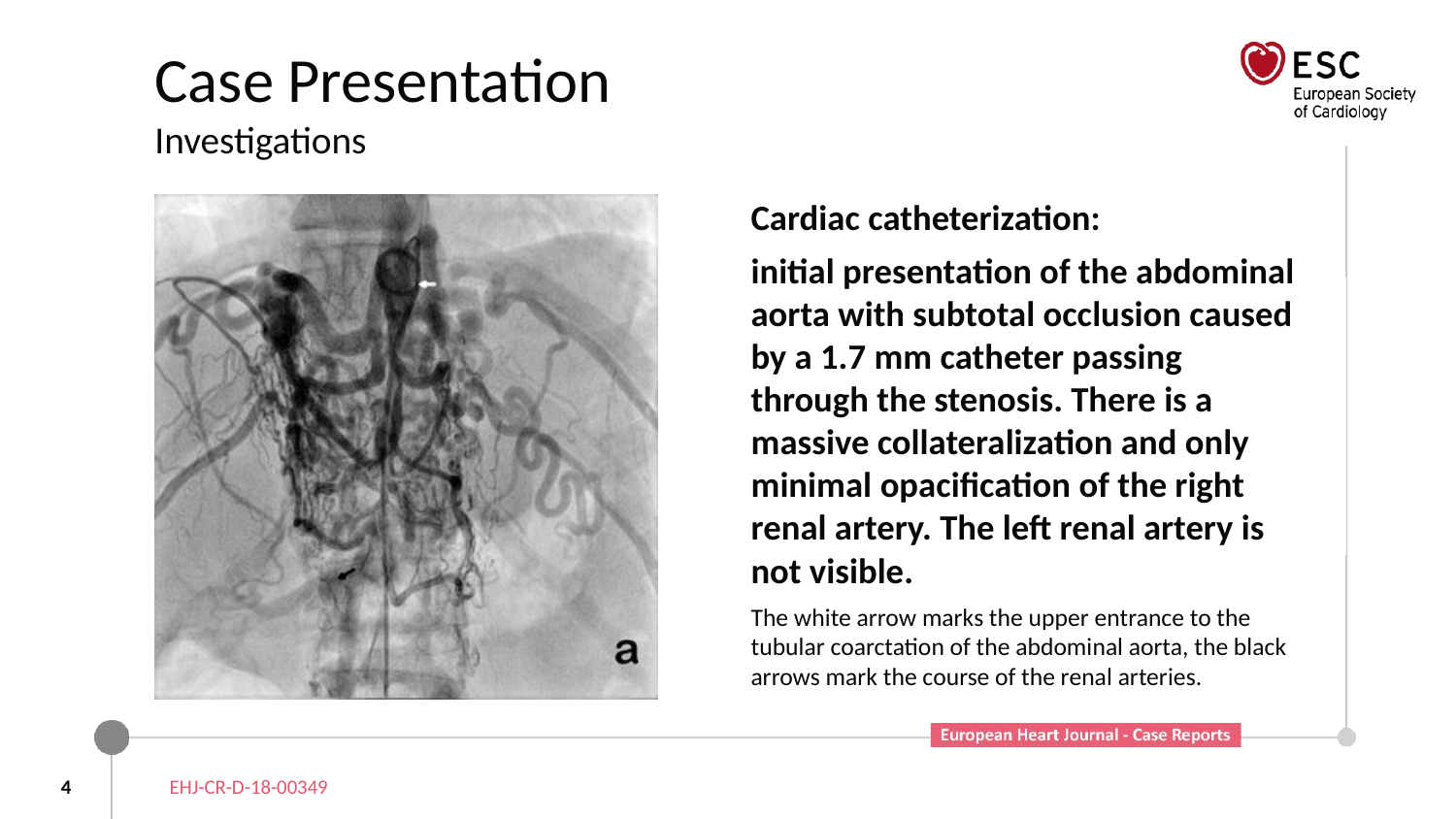

# Case Presentation Investigations
Cardiac catheterization:
initial presentation of the abdominal aorta with subtotal occlusion caused by a 1.7 mm catheter passing through the stenosis. There is a massive collateralization and only minimal opacification of the right renal artery. The left renal artery is not visible.
The white arrow marks the upper entrance to the tubular coarctation of the abdominal aorta, the black arrows mark the course of the renal arteries.
4
EHJ-CR-D-18-00349

## Slide 5
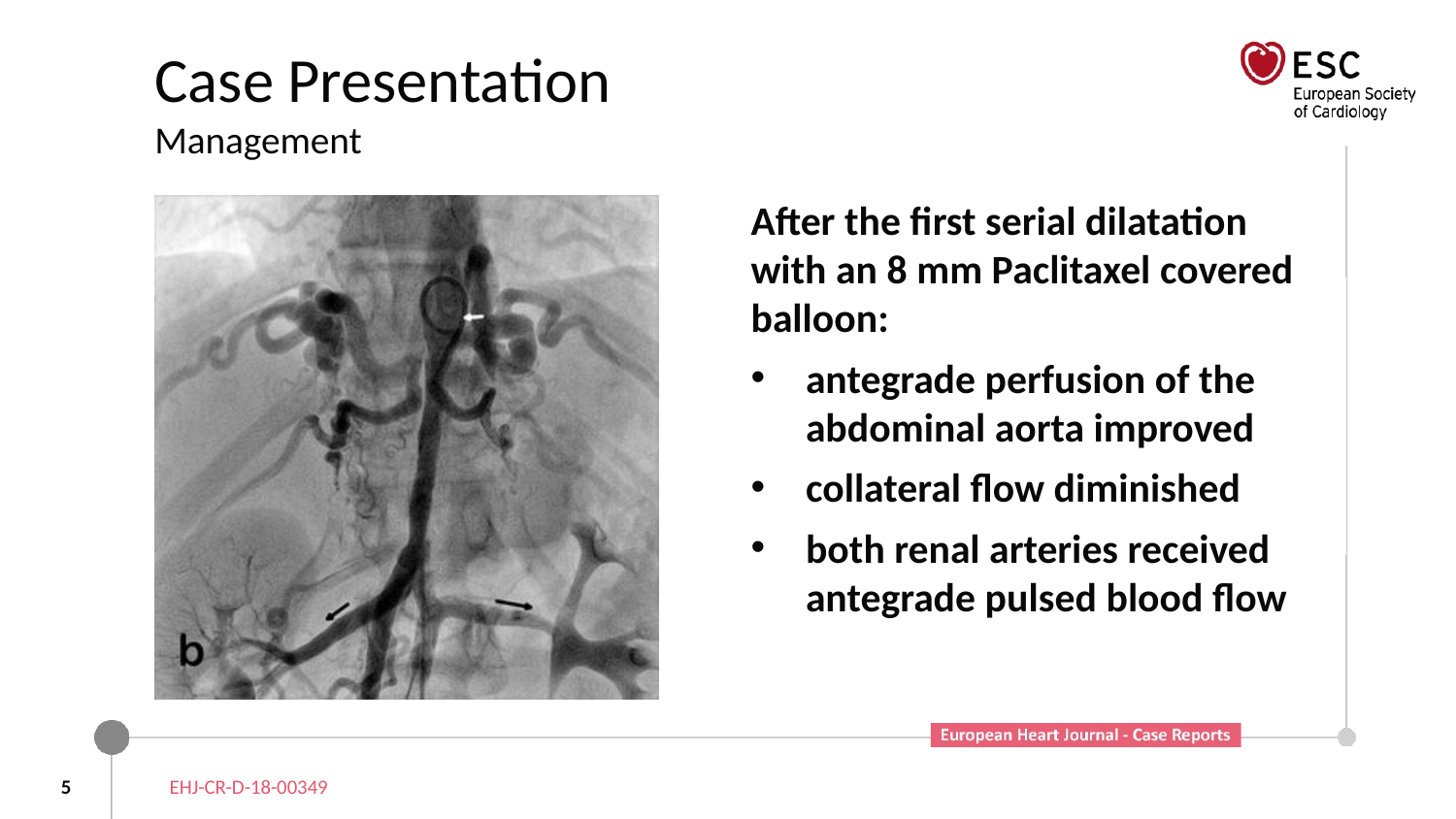

# Case Presentation Management
After the first serial dilatation with an 8 mm Paclitaxel covered balloon:
antegrade perfusion of the abdominal aorta improved
collateral flow diminished
both renal arteries received antegrade pulsed blood flow
5
EHJ-CR-D-18-00349

## Slide 6
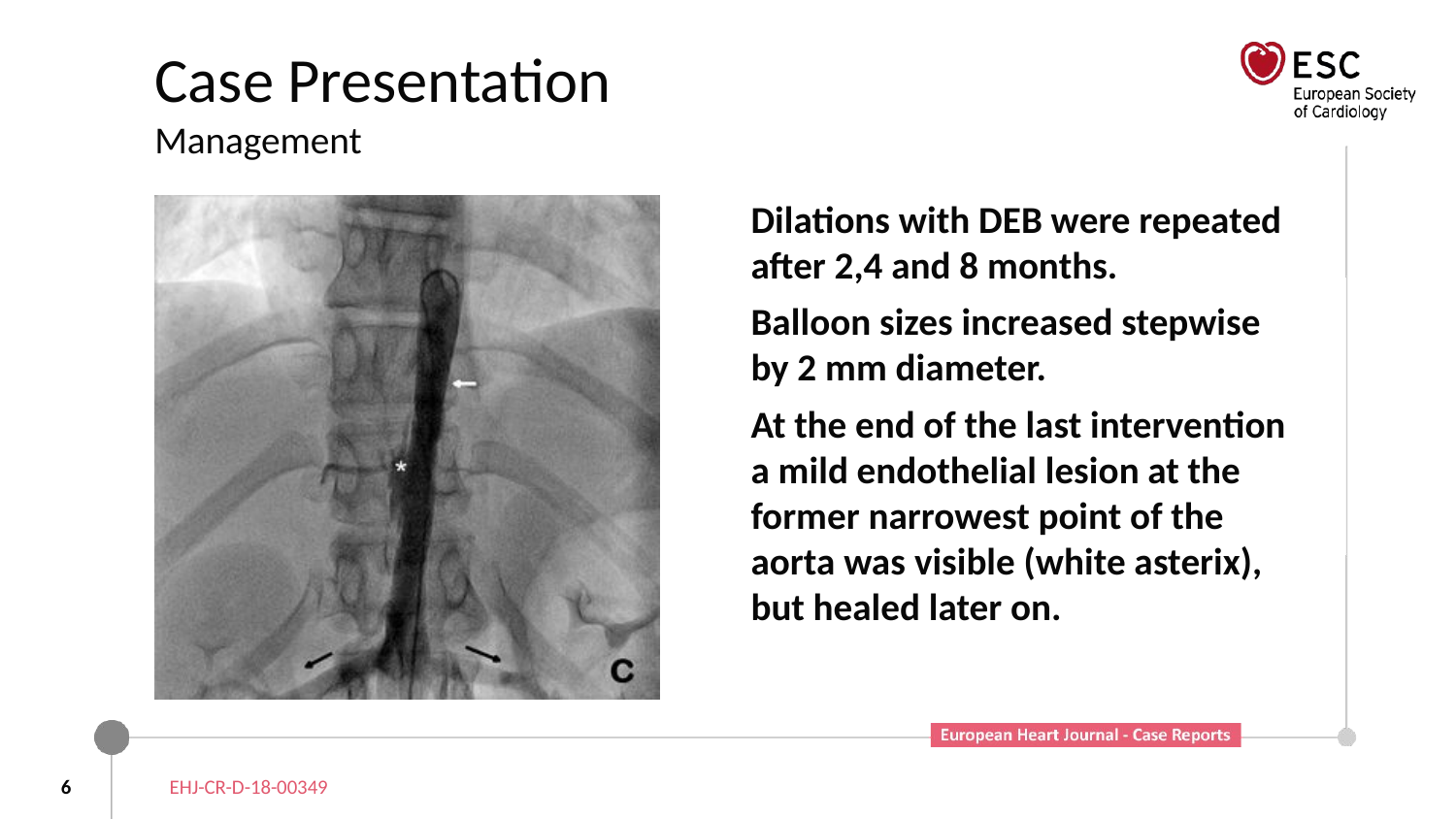

# Case Presentation Management
Dilations with DEB were repeated after 2,4 and 8 months.
Balloon sizes increased stepwise by 2 mm diameter.
At the end of the last intervention a mild endothelial lesion at the former narrowest point of the aorta was visible (white asterix), but healed later on.
6
EHJ-CR-D-18-00349

## Slide 7
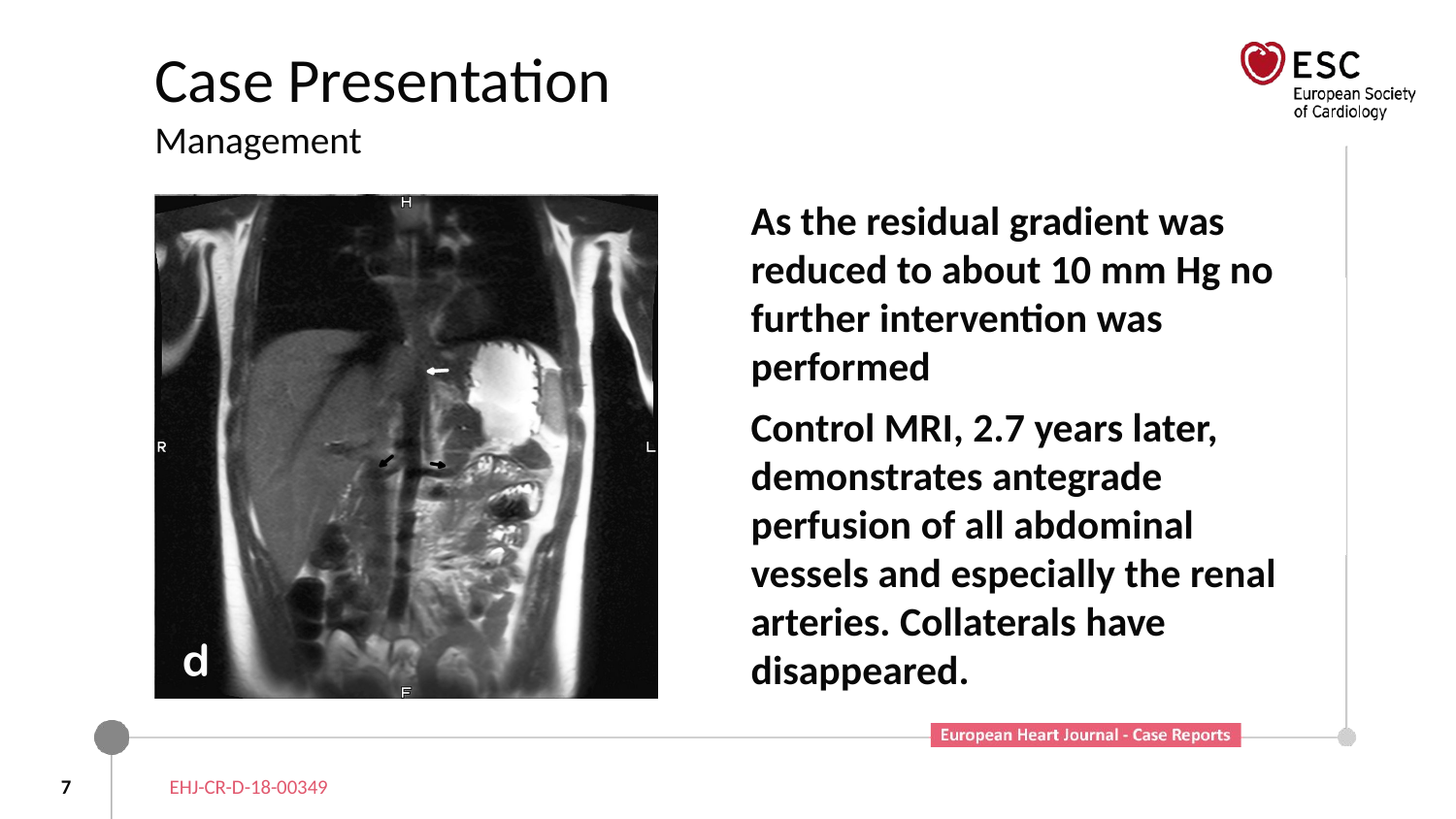

# Case Presentation Management
As the residual gradient was reduced to about 10 mm Hg no further intervention was performed
Control MRI, 2.7 years later, demonstrates antegrade perfusion of all abdominal vessels and especially the renal arteries. Collaterals have disappeared.
7
EHJ-CR-D-18-00349

## Slide 8
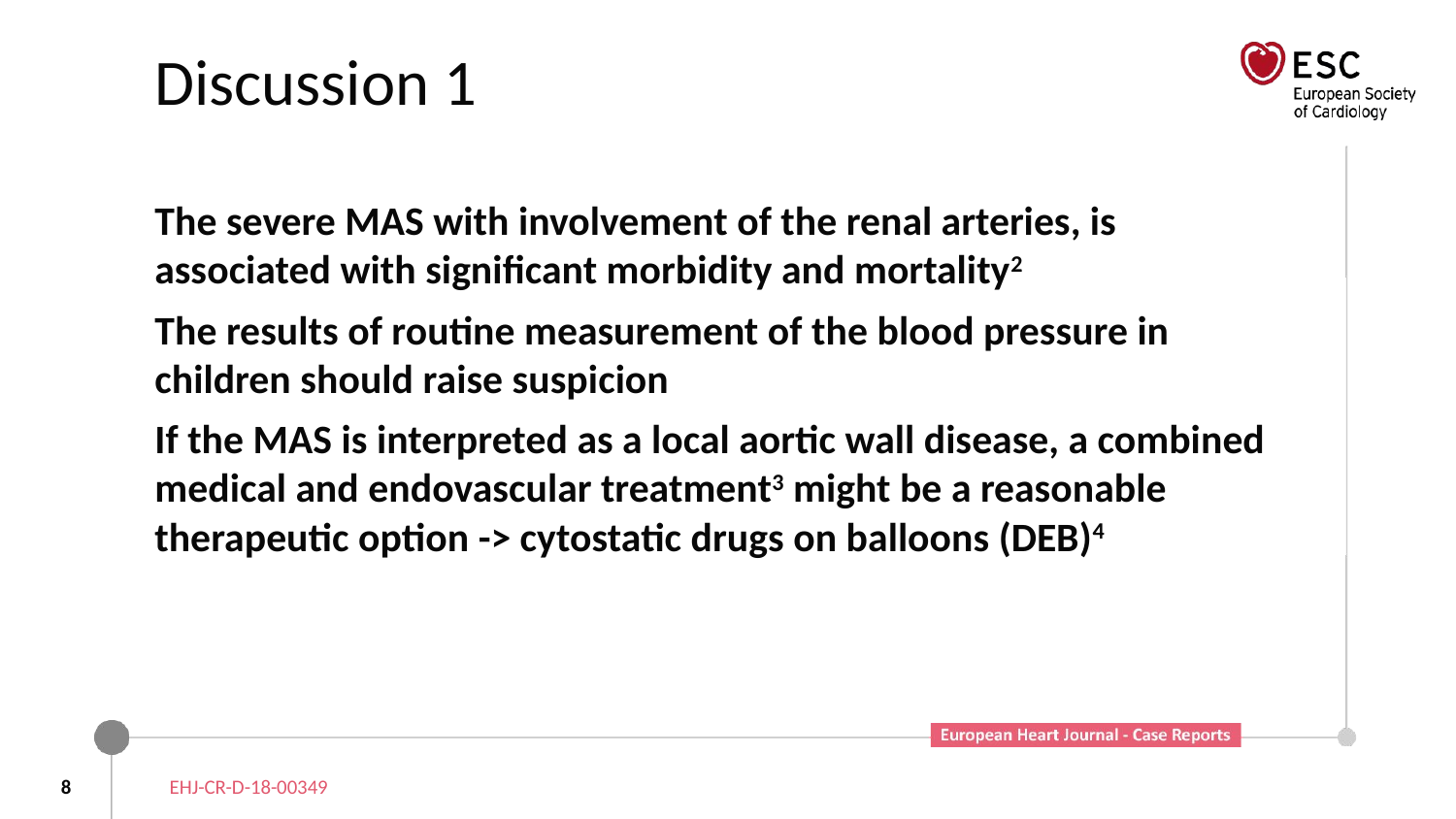

# Discussion 1
The severe MAS with involvement of the renal arteries, is associated with significant morbidity and mortality2
The results of routine measurement of the blood pressure in children should raise suspicion
If the MAS is interpreted as a local aortic wall disease, a combined medical and endovascular treatment3 might be a reasonable therapeutic option -> cytostatic drugs on balloons (DEB)4
8
EHJ-CR-D-18-00349

## Slide 9
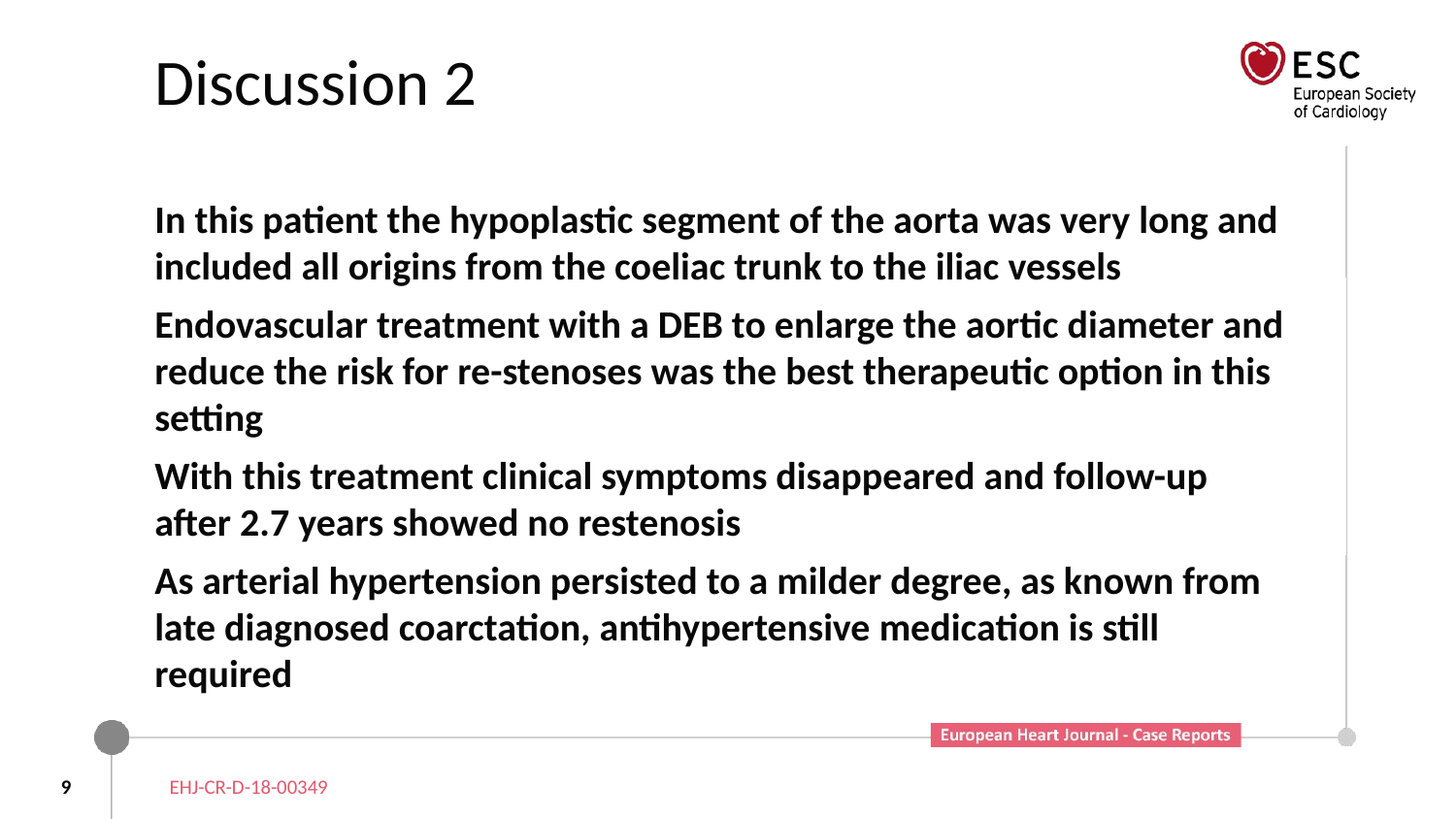

# Discussion 2
In this patient the hypoplastic segment of the aorta was very long and included all origins from the coeliac trunk to the iliac vessels
Endovascular treatment with a DEB to enlarge the aortic diameter and reduce the risk for re-stenoses was the best therapeutic option in this setting
With this treatment clinical symptoms disappeared and follow-up after 2.7 years showed no restenosis
As arterial hypertension persisted to a milder degree, as known from late diagnosed coarctation, antihypertensive medication is still required
9
EHJ-CR-D-18-00349

## Slide 10
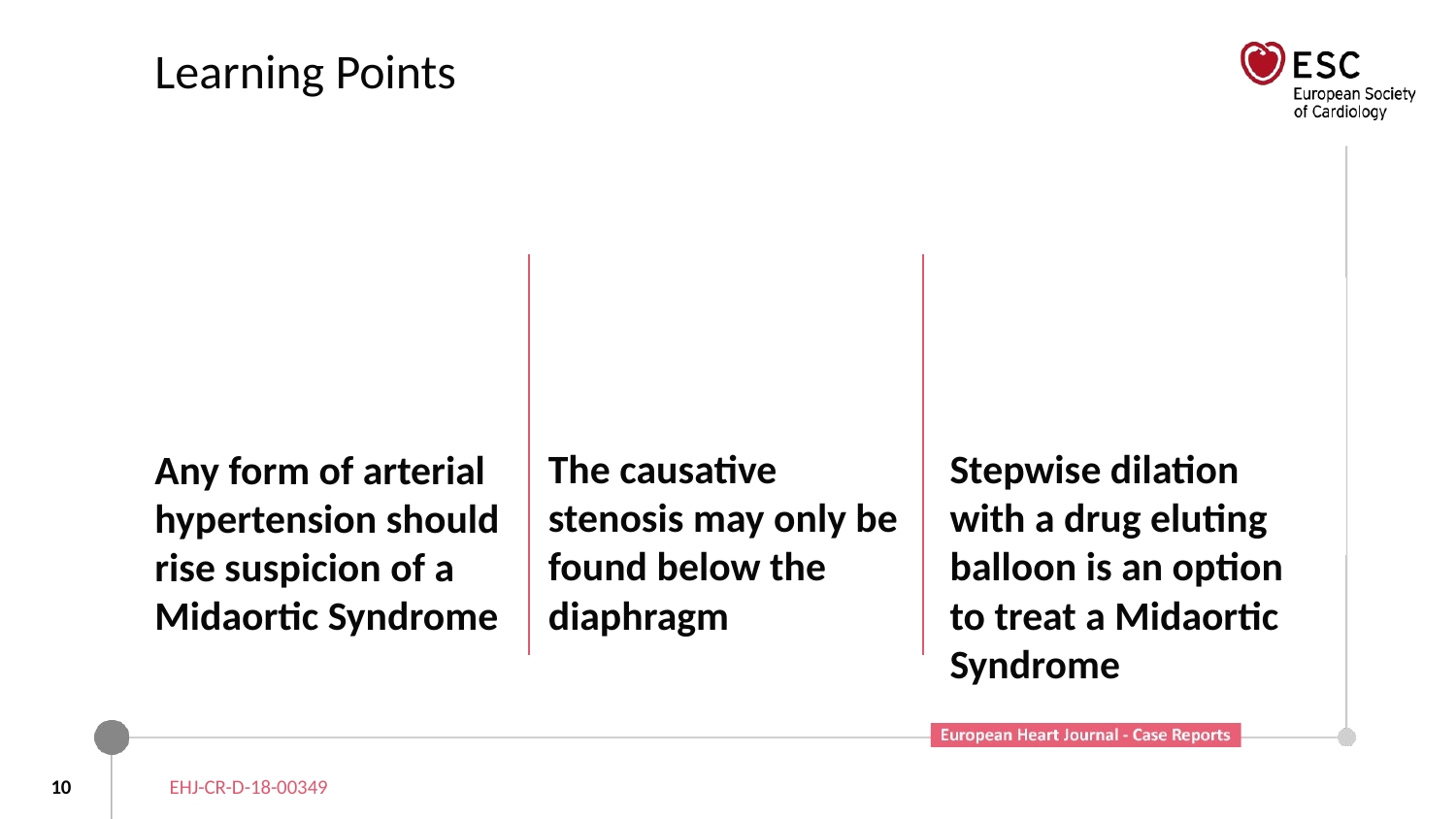

# Learning Points
Any form of arterial hypertension should rise suspicion of a Midaortic Syndrome
The causative stenosis may only be found below the diaphragm
Stepwise dilation with a drug eluting balloon is an option to treat a Midaortic Syndrome
10
EHJ-CR-D-18-00349

## Slide 11
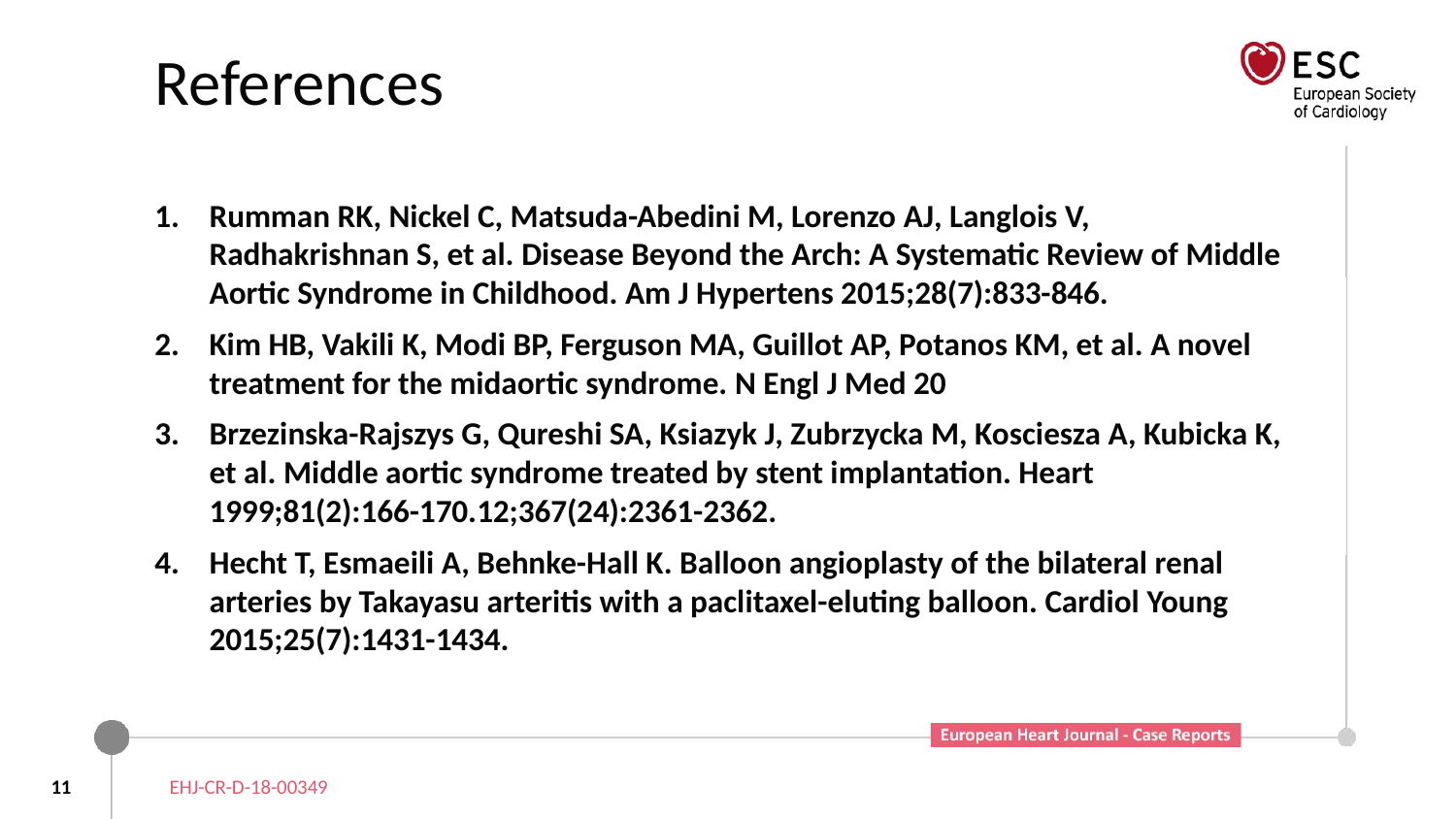

# References
Rumman RK, Nickel C, Matsuda-Abedini M, Lorenzo AJ, Langlois V, Radhakrishnan S, et al. Disease Beyond the Arch: A Systematic Review of Middle Aortic Syndrome in Childhood. Am J Hypertens 2015;28(7):833-846.
Kim HB, Vakili K, Modi BP, Ferguson MA, Guillot AP, Potanos KM, et al. A novel treatment for the midaortic syndrome. N Engl J Med 20
Brzezinska-Rajszys G, Qureshi SA, Ksiazyk J, Zubrzycka M, Kosciesza A, Kubicka K, et al. Middle aortic syndrome treated by stent implantation. Heart 1999;81(2):166-170.12;367(24):2361-2362.
Hecht T, Esmaeili A, Behnke-Hall K. Balloon angioplasty of the bilateral renal arteries by Takayasu arteritis with a paclitaxel-eluting balloon. Cardiol Young 2015;25(7):1431-1434.
11
EHJ-CR-D-18-00349
